# (Thio­cyanato-κ*N*)bis­(thio­semicarbazide-κ*S*)copper(I)

**DOI:** 10.1107/S1600536808013068

**Published:** 2008-05-10

**Authors:** Li Jia, Shouxin Ma, Dacheng Li

**Affiliations:** a LiaoCheng Vocational and Technical College, Liaocheng, Shandong 252000, People’s Republic of China; b Shandong Vocational Animal Science and Veterinary College, Weifang, Shandong 261000, People’s Republic of China; c School of Chemistry and Chemical Engineering, Liaocheng University, Shandong 252059, People’s Republic of China

## Abstract

In the title complex, [Cu(CH_5_N_3_S)_2_(NCS)], the non-H part of the mol­ecule is strictly planar, lying on the mirror plane at *y* = 0.25. The Cu atom lies at the centre of a triangle formed by the coordination of three monodentate groups, *viz*. two thio­semicarbazide ligands and one NCS^−^ anion. Weak inter­molecular N—H⋯S inter­actions generate a two-dimensional network.

## Related literature

For related thio­semicarbazide metal complexes, see: Capacchi *et al.* (1968 ▶). For related literature, see: Chattopadhyay *et al.* (1991 ▶).
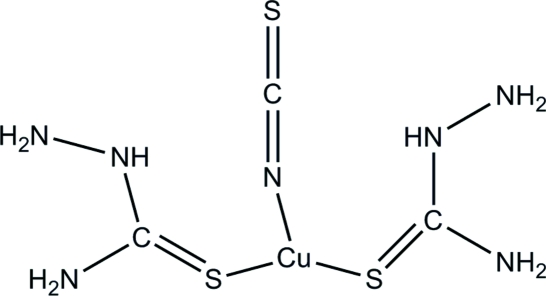


## Experimental

### 

#### Crystal data

[Cu(CH_5_N_3_S)_2_(NCS)]
*M* *_r_* = 303.90
Orthorhombic, 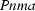

*a* = 11.488 (2) Å
*b* = 6.6085 (12) Å
*c* = 14.650 (3) Å
*V* = 1112.2 (4) Å^3^
*Z* = 4
Mo *K*α radiation
μ = 2.50 mm^−1^
*T* = 298 (2) K
0.38 × 0.27 × 0.24 mm

#### Data collection

Bruker SMART CCD area-detector diffractometer
Absorption correction: multi-scan (*SADABS*; Sheldrick, 1996 ▶) *T*_min_ = 0.44, *T*_max_ = 0.55
5593 measured reflections
1077 independent reflections
947 reflections with *I* > 2σ(*I*)
*R*_int_ = 0.021

#### Refinement

*R*[*F* ^2^ > 2σ(*F* ^2^)] = 0.029
*wR*(*F* ^2^) = 0.080
*S* = 1.05
1077 reflections
86 parameters
H-atom parameters constrained
Δρ_max_ = 0.67 e Å^−3^
Δρ_min_ = −0.54 e Å^−3^

### 

Data collection: *SMART* (Bruker, 1997 ▶); cell refinement: *SAINT* (Bruker, 1997 ▶); data reduction: *SAINT*; program(s) used to solve structure: *SHELXS97* (Sheldrick, 2008 ▶); program(s) used to refine structure: *SHELXL97* (Sheldrick, 2008 ▶); molecular graphics: *SHELXTL* (Sheldrick, 2008 ▶); software used to prepare material for publication: *SHELXTL*.

## Supplementary Material

Crystal structure: contains datablocks I, global. DOI: 10.1107/S1600536808013068/bg2183sup1.cif

Structure factors: contains datablocks I. DOI: 10.1107/S1600536808013068/bg2183Isup2.hkl

Additional supplementary materials:  crystallographic information; 3D view; checkCIF report

## Figures and Tables

**Table 1:** Hydrogen-bond geometry (Å, °)

*D*—H⋯*A*	*D*—H	H⋯*A*	*D*⋯*A*	*D*—H⋯*A*
N1—H1*A*⋯S3^i^	0.86	2.64	3.485 (3)	167
N1—H1*B*⋯N3^i^	0.86	2.14	2.998 (4)	177
N2—H2⋯N7	0.86	2.24	3.093 (4)	175
N3—H3*A*⋯S2^ii^	0.89	2.81	3.5481 (11)	141
N3—H3*B*⋯S2^iii^	0.89	2.72	3.5481 (11)	155
N4—H4*A*⋯S2^iv^	0.86	2.65	3.501 (3)	170
N4—H4*B*⋯N7	0.86	2.32	3.161 (4)	167
N5—H5⋯S3^v^	0.86	2.64	3.342 (3)	140
N6—H6*A*⋯S1^vi^	0.89	2.88	3.5144 (12)	130
N6—H6*B*⋯S1^vii^	0.89	2.75	3.5144 (11)	144

Symmetry codes: (i) 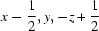; (ii) 
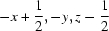
; (iii) 
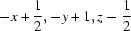
; (iv) 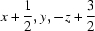; (v) 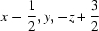; (vi) 
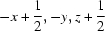
; (vii) 
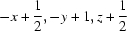
.

**Fig. 1.**
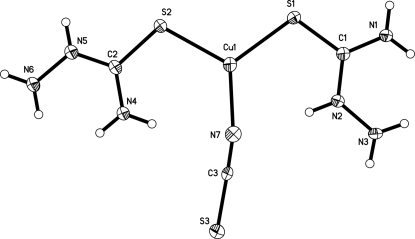
The crystal structure of the title compound showing the atomic numbering and 30% probability displacement ellipsoids. N-bound H atoms have been omitted for clarity.

## supplementary crystallographic information

### Comment

Thiosemicarbazide can behave as a chelating agent(Chattopadhyay *et al.*,1991) or as a monodentate ligand(Capacchi *et al.*, 1968). Among the different N,*S* donors studies,thiosemicarbazides and thiosemicarbazines are of special interest as both of these free ligands and their copper complexes exhibit a variety of biological activities including antitumour activity(Chattopadhyay *et al.*,1991). In this paper, Cu(CH_5_N_3_S)_2_(NCS) was synthesized by the reaction of CuCl_2_.6H_2_O, thiosemicarbazide and NaSCN at room temperature and the structure of the resulting complex is presented herein. The non-H part of the molecule is strictly planar, lying on the mirror plane at y=0.25. The Cu atom lies at the centre of a planar triangle formed by coordination of three monodentate groups, two thiosemicarbazide ligands and one NCS anion (Fig. 1). The Cu—S bond length (2.2415 (13) Å) is comparable to those in [Cu(SC(NH_2_)NHNH_2_)Cl_2_] (2.266 (1) Å,Chattopadhyay *et al.*, 1991), while the Cu—N bond is shorter than the corresponding value therein (2.002 (4)Å).

In the crystal structure, weak intermolecular C—H···S interactions determine a two-dimensional network.

### Experimental

Copper chloride dihydrate (0.3 mmol 51.2 mg) was dissolved in absolute methanol (10 ml), and was added dropwise to a solution of an equate ligand thiosemicarbazide in MeOH (10 ml). The solution was stirred for 10 minutes, then NaSCN was added. The solution became bottle-green. The mother liquid was placed at room temperature, and single crystals were obtained on standing. Elemental analysis for C_3_H_10_Cu N_7_S_3_ calculated: C 36.03, H 10.08, N %; found: C 36.12, H 10.26, N 98.05%.

### Refinement

All H atoms were placed geometrically and treated as riding on their parent atoms with N—H 0.86 Å (thiosemicarbazide) [*U*_iso_(H) = 1.2*U*_eq_(N)].

The copper atom presents an abnormally elongated displacement ellipsoid perpendicular to the mirror plane, suggeesting some kind of disorder around its (average) symmetric position.

### Crystal data

**Table:**

[Cu(CH_5_N_3_S)_2_(NCS)]	*F*(000) = 616
*M**_r_* = 303.90	*D*_x_ = 1.815 Mg m^−^^3^
Orthorhombic, *P**n**m**a*	Mo *K*α radiation, λ = 0.71073 Å
Hall symbol: -P 2ac 2n	Cell parameters from 3130 reflections
*a* = 11.488 (2) Å	θ = 2.3–28.2°
*b* = 6.6085 (12) Å	µ = 2.50 mm^−^^1^
*c* = 14.650 (3) Å	*T* = 298 K
*V* = 1112.2 (4) Å^3^	Block, black
*Z* = 4	0.38 × 0.27 × 0.24 mm

### Data collection

**Table:**

Bruker SMART CCD area-detector diffractometer	1077 independent reflections
Radiation source: fine-focus sealed tube	947 reflections with *I* > 2σ(*I*)
graphite	*R*_int_ = 0.021
φ and ω scans	θ_max_ = 25.0°, θ_min_ = 2.3°
Absorption correction: multi-scan (*SADABS*; Sheldrick, 1996)	*h* = −13→13
*T*_min_ = 0.44, *T*_max_ = 0.55	*k* = −7→7
5593 measured reflections	*l* = −17→10

### Refinement

**Table:**

Refinement on *F*^2^	Secondary atom site location: difference Fourier map
Least-squares matrix: full	Hydrogen site location: inferred from neighbouring sites
*R*[*F*^2^ > 2σ(*F*^2^)] = 0.029	H-atom parameters constrained
*wR*(*F*^2^) = 0.080	*w* = 1/[σ^2^(*F*_o_^2^) + (0.0469*P*)^2^ + 0.712*P*] where *P* = (*F*_o_^2^ + 2*F*_c_^2^)/3
*S* = 1.05	(Δ/σ)_max_ < 0.001
1077 reflections	Δρ_max_ = 0.67 e Å^−^^3^
86 parameters	Δρ_min_ = −0.54 e Å^−^^3^
0 restraints	Extinction correction: *SHELXL97* (Sheldrick, 2008), Fc^*^=kFc[1+0.001xFc^2^λ^3^/sin(2θ)]^-1/4^
Primary atom site location: structure-invariant direct methods	Extinction coefficient: 0.0072 (9)

### Special details

**Table:**

Geometry. All e.s.d.'s (except the e.s.d. in the dihedral angle between two l.s. planes) are estimated using the full covariance matrix. The cell e.s.d.'s are taken into account individually in the estimation of e.s.d.'s in distances, angles and torsion angles; correlations between e.s.d.'s in cell parameters are only used when they are defined by crystal symmetry. An approximate (isotropic) treatment of cell e.s.d.'s is used for estimating e.s.d.'s involving l.s. planes.
Refinement. Refinement of *F*^2^ against ALL reflections. The weighted *R*-factor *wR* and goodness of fit *S* are based on *F*^2^, conventional *R*-factors *R* are based on *F*, with *F* set to zero for negative *F*^2^. The threshold expression of *F*^2^ > σ(*F*^2^) is used only for calculating *R*-factors(gt) *etc*. and is not relevant to the choice of reflections for refinement. *R*-factors based on *F*^2^ are statistically about twice as large as those based on *F*, and *R*- factors based on ALL data will be even larger.

### Fractional atomic coordinates and isotropic or equivalent isotropic displacement parameters (Å^2^)

**Table:**

	*x*	*y*	*z*	*U*_iso_*/*U*_eq_	Occ. (<1)
Cu1	0.28439 (4)	0.2500	0.53244 (3)	0.0689 (3)	
N1	0.1200 (2)	0.2500	0.25507 (19)	0.0338 (6)	
H1A	0.1489	0.2500	0.2009	0.041*	
H1B	0.0458	0.2500	0.2625	0.041*	
N2	0.3047 (2)	0.2500	0.31376 (19)	0.0361 (7)	
H2	0.3483	0.2500	0.3615	0.043*	
N3	0.3600 (2)	0.2500	0.22780 (18)	0.0348 (6)	
H3A	0.3244	0.1616	0.1914	0.052*	0.50
H3B	0.3555	0.3732	0.2035	0.052*	0.50
N4	0.4655 (2)	0.2500	0.7081 (2)	0.0457 (8)	
H4A	0.5262	0.2500	0.7423	0.055*	
H4B	0.4729	0.2500	0.6496	0.055*	
N5	0.3516 (2)	0.2500	0.83438 (18)	0.0349 (7)	
H5	0.2839	0.2500	0.8594	0.042*	
N6	0.4526 (2)	0.2500	0.88959 (19)	0.0414 (7)	
H6A	0.4426	0.1645	0.9359	0.062*	0.50
H6B	0.4647	0.3741	0.9112	0.062*	0.50
N7	0.4475 (3)	0.2500	0.4928 (2)	0.0415 (7)	
S1	0.13368 (7)	0.2500	0.43541 (6)	0.0335 (2)	
S2	0.23512 (7)	0.2500	0.68064 (5)	0.0318 (2)	
S3	0.68866 (8)	0.2500	0.47665 (7)	0.0528 (3)	
C1	0.1893 (3)	0.2500	0.3264 (2)	0.0283 (7)	
C2	0.3611 (3)	0.2500	0.7450 (2)	0.0286 (7)	
C3	0.5450 (3)	0.2500	0.4851 (2)	0.0278 (7)	

### Atomic displacement parameters (Å^2^)

**Table:**

	*U*^11^	*U*^22^	*U*^33^	*U*^12^	*U*^13^	*U*^23^
Cu1	0.0292 (3)	0.1520 (7)	0.0256 (3)	0.000	−0.00124 (17)	0.000
N1	0.0205 (13)	0.0557 (17)	0.0252 (13)	0.000	−0.0003 (10)	0.000
N2	0.0214 (13)	0.0632 (18)	0.0237 (14)	0.000	−0.0002 (11)	0.000
N3	0.0254 (13)	0.0517 (17)	0.0274 (15)	0.000	0.0048 (11)	0.000
N4	0.0261 (15)	0.088 (2)	0.0236 (14)	0.000	−0.0024 (11)	0.000
N5	0.0260 (13)	0.0529 (17)	0.0259 (15)	0.000	−0.0030 (11)	0.000
N6	0.0339 (15)	0.0617 (19)	0.0285 (15)	0.000	−0.0088 (12)	0.000
N7	0.0338 (18)	0.0549 (19)	0.0357 (16)	0.000	0.0011 (13)	0.000
S1	0.0242 (4)	0.0521 (5)	0.0242 (4)	0.000	0.0021 (3)	0.000
S2	0.0232 (4)	0.0477 (5)	0.0243 (4)	0.000	−0.0017 (3)	0.000
S3	0.0279 (5)	0.0971 (8)	0.0333 (5)	0.000	−0.0009 (4)	0.000
C1	0.0242 (15)	0.0345 (16)	0.0263 (16)	0.000	−0.0003 (12)	0.000
C2	0.0256 (15)	0.0335 (16)	0.0265 (16)	0.000	−0.0038 (12)	0.000
C3	0.0286 (18)	0.0395 (18)	0.0152 (14)	0.000	0.0017 (12)	0.000

### Geometric parameters (Å, °)

**Table:**

Cu1—N7	1.962 (3)	N4—H4A	0.8600
Cu1—S1	2.2401 (10)	N4—H4B	0.8600
Cu1—S2	2.2437 (10)	N5—C2	1.314 (4)
N1—C1	1.313 (4)	N5—N6	1.414 (4)
N1—H1A	0.8600	N5—H5	0.8600
N1—H1B	0.8600	N6—H6A	0.8900
N2—C1	1.339 (4)	N6—H6B	0.8900
N2—N3	1.410 (4)	N7—C3	1.126 (4)
N2—H2	0.8600	S1—C1	1.721 (3)
N3—H3A	0.8900	S2—C2	1.727 (3)
N3—H3B	0.8900	S3—C3	1.655 (3)
N4—C2	1.315 (4)		
			
N7—Cu1—S1	123.38 (10)	C2—N5—H5	119.9
N7—Cu1—S2	121.85 (10)	N6—N5—H5	119.9
S1—Cu1—S2	114.77 (4)	N5—N6—H6A	109.3
C1—N1—H1A	120.0	N5—N6—H6B	109.4
C1—N1—H1B	120.0	H6A—N6—H6B	109.5
H1A—N1—H1B	120.0	C3—N7—Cu1	168.5 (3)
C1—N2—N3	124.7 (3)	C1—S1—Cu1	107.58 (11)
C1—N2—H2	117.7	C2—S2—Cu1	108.46 (11)
N3—N2—H2	117.7	N1—C1—N2	119.4 (3)
N2—N3—H3A	109.2	N1—C1—S1	120.9 (2)
N2—N3—H3B	109.4	N2—C1—S1	119.7 (2)
H3A—N3—H3B	109.5	N5—C2—N4	119.0 (3)
C2—N4—H4A	120.0	N5—C2—S2	118.3 (2)
C2—N4—H4B	120.0	N4—C2—S2	122.7 (3)
H4A—N4—H4B	120.0	N7—C3—S3	178.6 (3)
C2—N5—N6	120.1 (3)		

### Hydrogen-bond geometry (Å, °)

**Table:**

*D*—H···*A*	*D*—H	H···*A*	*D*···*A*	*D*—H···*A*
N1—H1A···S3^i^	0.86	2.64	3.485 (3)	167.
N1—H1B···N3^i^	0.86	2.14	2.998 (4)	177.
N2—H2···N7	0.86	2.24	3.093 (4)	175.
N3—H3A···S2^ii^	0.89	2.81	3.5481 (11)	141.
N3—H3B···S2^iii^	0.89	2.72	3.5481 (11)	155.
N4—H4A···S2^iv^	0.86	2.65	3.501 (3)	170.
N4—H4B···N7	0.86	2.32	3.161 (4)	167.
N5—H5···S3^v^	0.86	2.64	3.342 (3)	140.
N6—H6A···S1^vi^	0.89	2.88	3.5144 (12)	130.
N6—H6B···S1^vii^	0.89	2.75	3.5144 (11)	144.

Symmetry codes: (i) *x*−1/2, *y*, −*z*+1/2; (ii) −*x*+1/2, −*y*, *z*−1/2; (iii) −*x*+1/2, −*y*+1, *z*−1/2; (iv) *x*+1/2, *y*, −*z*+3/2; (v) *x*−1/2, *y*, −*z*+3/2; (vi) −*x*+1/2, −*y*, *z*+1/2; (vii) −*x*+1/2, −*y*+1, *z*+1/2.
